# Interactions of *Streptococcus suis* serotype 9 with host cells and role of the capsular polysaccharide: Comparison with serotypes 2 and 14

**DOI:** 10.1371/journal.pone.0223864

**Published:** 2019-10-10

**Authors:** Jean-Philippe Auger, Servane Payen, David Roy, Audrey Dumesnil, Mariela Segura, Marcelo Gottschalk

**Affiliations:** Research Group on Infectious Diseases in Production Animals (GREMIP) and Swine and Poultry Infectious Diseases Research Center (CRIPA), Department of Pathology and Microbiology, Faculty of Veterinary Medicine, Université de Montréal, Saint-Hyacinthe, Quebec, Canada; All India Institute of Medical Sciences, INDIA

## Abstract

*Streptococcus suis* is an important porcine bacterial pathogen and a zoonotic agent responsible for sudden death, septic shock and meningitis, of which serotype 2 is the most widespread, with serotype 14 also causing infections in humans in South-East Asia. Knowledge of its pathogenesis and virulence are almost exclusively based on these two serotypes. Though serotype 9 is responsible for the greatest number of porcine cases in Spain, the Netherlands and Germany, very little information is currently available regarding this serotype. Of the different virulence factors, the capsular polysaccharide (CPS) is required for *S*. *suis* virulence as it promotes resistance to phagocytosis and killing and masks surface components responsible for host cell activation. However, these roles have been described for serotypes 2 and 14, whose CPSs are structurally and compositionally similar, both containing sialic acid. Consequently, we evaluated herein the interactions of serotype 9 with host cells and the role of its CPS, which greatly differs from those of serotypes 2 and 14. Results demonstrated that serotype 9 adhesion to but not invasion of respiratory epithelial cells was greater than that of serotypes 2 and 14. Furthermore serotype 9 was more internalized by macrophages but equally resistant to whole blood killing. Though recognition of serotypes 2, 9 and 14 by DCs required MyD88-dependent signaling, *in vitro* pro-inflammatory mediator production induced by serotype 9 was much lower. *In vivo*, however, serotype 9 causes an exacerbated inflammatory response, which combined with persistent bacterial presence, is probably responsible for host death during the systemic infection. Though presence of the serotype 9 CPS masks surface components less efficiently than those of serotypes 2 and 14, the serotype 9 CPS remains critical for virulence as it is required for survival in blood and development of clinical disease, and this regardless of its unique composition and structure.

## Introduction

*Streptococcus suis* is an important encapsulated bacterial pathogen of young piglets and a zoonotic agent causing a variety of pathologies including sudden death (pigs), septic shock (humans) and meningitis (both species) [1]. Classification is based on serotyping as defined by the antigenicity of the capsular polysaccharide (CPS) or by the presence of serotype-specific genes [1]. Of the thirty-five described serotypes, serotype 2 is the most widespread and virulent, being responsible for the majority of porcine and human cases of infection [2]. Alongside, serotype 14 is an emerging threat to human health in South-East Asia [2]. Other than a few reports on serotype 14 [3, 4], serotype 2 remains by far the most studied serotype, with current understanding of the *S*. *suis* pathogenesis and virulence almost exclusively based on it [5, 6]. As such, our knowledge regarding other serotypes remains limited. Together with serotypes 2 and 14, serotype 9 has emerged in Europe in recent years and is presently responsible for the greatest number of porcine cases of *S*. *suis* infection in Spain, the Netherlands and Germany [2]. Furthermore, its prevalence in China [7] and Canada [8] has significantly increased, with the first human case being reported in Thailand [9]. Nevertheless, very few studies have addressed the interactions of this serotype with host cells [10–12].

Though a variety of virulence factors have been described for *S*. *suis*, the CPS is considered to be one of its few truly critical virulence factors and is implicated in a multitude of functions [5, 6]. These include, for example, resistance to phagocytosis and killing by innate immune cells [13–18] and masking of surface components responsible for host cell activation [17–19]. Indeed, presence of CPS interferes with recognition of *S*. *suis* by Toll-like receptors (TLRs), a family of evolutionarily conserved membrane-associated innate immune receptors that mainly signal via myeloid differentiation primary response 88 (MyD88) [19, 20]. Moreover, studies using experimental animal infection models have demonstrated that the CPS is required for survival in blood [18, 21, 22]. Alongside, it was recently demonstrated that *S*. *suis* can modulate the presence of its CPS within the host [23], a mechanism that could participate in host cell adhesion and invasion since these functions are hampered by its presence [5].

However, these roles have been described for serotype 2 and, more recently, for serotype 14 [3, 16, 18, 22]. Moreover, presence of CPS was also reported to confer anti-phagocytic properties to serotypes 1 and 1/2 [24]. Though certain structural and composition differences exist between the CPSs of these four serotypes, they are minimal: the serotype 14 and 1 and serotype 2 and 1/2 CPSs, respectively, only differ by the substitution of a galactose to a *N*-acetylgalactosamine resulting from a single nucleotide polymorphism in the glycosyltransferase CpsK [24–27]. In fact, switching CPS expression between serotypes 1 and 14 or between serotypes 1/2 and 2 had limited impact on virulence due to similar anti-phagocytic properties [24]. Furthermore, the CPSs of these four serotypes are characterized by the presence of a sialic acid (*N*-acetylneuraminic acid) sidechain [25–27]. Sialic acid is commonly present in host cells and confers important properties to the cell surface [28]. Though different pathogens have evolved to express sialic acid at their surface, *S*. *suis* is one of only two sialylated Gram-positive bacteria, the other being Group B *Streptococcus* (GBS) [28]. Importantly, presence of sialic acid in the GBS CPS is associated with modulation of immune cell activation [29, 30]. Unfortunately, it has not yet been possible to evaluate the role of sialic acid in *S*. *suis* pathogenesis since deletion of the sialyltransferase or sialic acid synthesis genes results in complete non-encapsulation, while mutations blocking its assembly are lethal due to an accumulation of intracellular sialic acid [18, 21, 31, 32]. As such, knowledge regarding the role of *S*. *suis* CPS in absence of sialic acid remains unknown. Interestingly, the composition and structure of the serotype 9 CPS was recently described, greatly differs from that of serotypes 2 and 14 and does not contain sialic acid [33]. Consequently, given the lack of information regarding the serotype 9 pathogenesis, its interactions with host cells and the role of its CPS were evaluated in comparison to the well-characterized serotypes 2 and 14.

## Materials and methods

### Ethics statement

This study was carried out in accordance with the recommendations of the guidelines and policies of the Canadian Council on Animal Care and the principles set forth in the Guide for the Care and Use of Laboratory Animals. The protocols and procedures were approved by the Animal Welfare Committee of the Université de Montréal (permit number Rech-1570).

### Bacterial strains and growth conditions

The encapsulated wild-type *S*. *suis* serotype 2, 9, and 14 strains used in this study, and their non-encapsulated isogenic mutants are listed in Table 1. As previously described, the serotype 9 1135776 strain was isolated from a diseased pig in Canada and belongs to sequence type 788 [34]. *S*. *suis* strains were cultured in Todd Hewitt broth (THB; Becton Dickinson, Mississauga, ON, Canada). For *in vitro* cell culture assays, bacteria were prepared as previously described [17, 35] and resuspended in cell culture medium. For experimental infections, early stationary phase bacteria were washed twice in phosphate-buffered saline pH 7.4 and resuspended in THB [36–38]. Bacterial cultures were appropriately diluted and plated on THB agar (THA) to accurately determine bacterial concentrations. The *Escherichia coli* strain and different plasmids used in this study are also listed in Table 1. When needed, antibiotics (Sigma-Aldrich, Oakville, ON, Canada) were added to the media at the following concentrations: for *S*. *suis*, spectinomycin at 100 μg/mL; for *E*. *coli*, kanamycin and spectinomycin at 50 μg/mL and ampicillin at 100 μg/mL.

**Table 1:** List of strains and plasmids used in this study.

Strain or plasmid	Characteristics	Reference
***Streptococcus suis***		
P1/7	Virulent serotype 2 (S2) strain isolated from a case of pig meningitis in the United Kingdom	[39]
P1/7Δ*cpsF*	Non-encapsulated isogenic mutant derived from P1/7; in frame deletion of *cpsF* gene	[17]
DAN13730	Virulent serotype 14 (S14) strain isolated from a human case in the Netherlands	[40]
DAN13730Δ*cpsB*	Non-encapsulated isogenic mutant derived from DAN13730; in frame deletion of *cpsB* gene	[3]
1135776	Virulent serotype 9 (S9) strain isolated from a diseased pig in Canada	[12]
1135776Δ*cpsG*	Non-encapsulated isogenic mutant derived from 1135776; in frame deletion of *cpsG* glycosyltransferase gene	This study
***Escherichia coli***		
TOP10	F^-^ mrcA Δ(mrr-hsdRMS-mcrBC) φ80 lacZΔM15 ΔlacX74 recA1 araD139 Δ(ara-leu) 7697 galU galK rpsL (Str^R^) endA1 nupG	Invitrogen
**Plasmids**		
pCR2.1	Ap^r^, Km^r^, pUC *ori*, *lac*ZΔM15	Invitrogen
pSET4s	Spc^r^, pUC *ori*, thermosensitive pG+host3 *ori*, *lac*ZΔM15	[41]
p4Δ*cpsG*	pSET-4s carrying the construct for *cpsG* allelic replacement	This study

### DNA manipulations

Genomic DNA was extracted from the *S*. *suis* serotype 9 1135776 strain using InstaGene Matrix solution (BioRad Laboratories, Hercules, CA, USA). Extraction and preparations of recombinant plasmids were carried out using the QIAprep Spin Miniprep Kit (Qiagen, Valencia, CA, USA). Restriction enzymes and DNA-modifying enzymes (Fisher Scientific, Ottawa, ON, Canada) were used according to the manufacturer’s recommendations. Oligonucleotide primers (Table 2) were obtained from Integrated DNA Technologies (Coralville, IA, USA) and PCRs carried out with the iProof proofreading DNA polymerase (BioRad Laboratories, Mississauga, ON, Canada) or the Taq DNA polymerase (Qiagen). Amplification products were purified using the QIAquick PCR Purification Kit (Qiagen) and sequenced using an ABI 310 Automated DNA Sequencer and ABI PRISM Dye Terminator Cycle Sequencing Kit (Applied Biosystems, Carlsbad, CA, USA).

**Table 2:** List of oligonucleotide primers used in this study.

Name	Sequence (5’– 3’)
*cpsG*-1	GGTAAGATTGAGTTGGTCC
*cpsG*-2	CTGATTGAGTGGCCCATCCTC
*cpsG*-3	GCAAACATTGATGAAACACT
*cpsG*-4	ATGAGATATGATGGCAAGCC
*cpsG*-5	GCGCGAATTCGTCTTGGATATGGGCGAGCCAG
*cpsG*-6	TCCATAAATGAGTTTTTCCCTAAGAAACTC
*cpsG*-7	GAGTTTCTTAGGGAAAAACTCATTTATGGA
*cpsG*-8	CGCGGAATTCATCATCGTCATCCTTCATTGC

Restriction sites are underlined

### Construction of the serotype 9 non-encapsulated isogenic mutant

The serotype 2 and 14 non-encapsulated isogenic mutants P1/7Δ*cpsF* and DAN13730Δ*cpsB* were previously constructed and characterized by our laboratory [3, 17]. Precise in-frame deletion of *cpsG* gene, encoding a glycosyltransferase, from 1135776 strain was constructed using splicing-by-overlap-extension PCRs as previously described [42, 43]. Overlapping PCR products were cloned into pCR2.1 (Invitrogen, Burlington, ON, Canada), extracted with EcoRI, recloned into the thermosensitive *E*. *coli*–*S*. *suis* shuttle plasmid pSET4s, and digested with the same enzyme, giving rise to the knockout vector p4Δ*cpsG*. Electroporation of the serotype 9 wild-type 1135776 strain and procedures for isolation of the mutants were previously described [41]. Allelic replacement was confirmed by PCR (S1 Appendix) and DNA sequencing analyses. Amplification products were purified with the QIAgen PCR Purification Kit (Qiagen) and sequenced as described above. Growth of the mutant strain was similar to that of its wild-type parental strain (S2 Appendix).

### Bacterial surface hydrophobicity assay

Relative surface hydrophobicity of the *S*. *suis* wild-type strains and non-encapsulated mutants was determined by measuring adsorption to *n*-hexadecane as previously described [18].

### Transmission electron microscopy

Unless otherwise indicated, chemicals were purchased from Sigma-Aldrich. Transmission electron microscopy was carried out as previously described [35, 44]. Briefly, bacteria were grown to mid-logarithmic phase and washed in 0.1 M cacodylate buffer pH 7.3 (Canemco & Marivac, Canton de Gore, QC) containing 2.5% glutaraldehyde and 0.05% ruthenium red. Ferritin was added to a final concentration of 1 mg/mL and incubated for 30 min at room temperature. Cells were then immobilized in 3% agar in 0.1M cacodylate buffer pH 7.3, washed five times in cacodylate buffer containing 0.05% ruthenium red, and fixed in 2% osmium tetroxide for 2 h at room temperature. Afterwards, samples were washed with water every 20 min for 2 h to remove osmium tetroxide and dehydrated in an increasing graded series of acetone. Specimens were then washed twice in propylene oxide and embedded in Spurr low-viscosity resin (Electron Microscopy Sciences, Hatfield, PA, USA). Thin sections were post-stained with uranyl acetate and lead citrate and examined using a transmission electron microscope at 80 kV (Hitachi model HT7770, Chiyoda, Tokyo, Japan).

### NPTr epithelial cell culture and adhesion and invasion assays

The porcine tracheal epithelial NPTr cell line was used and cultured until confluence as previously described [45]. Cells were infected with 1 x 10^6^ CFU/well (multiplicity of infection [MOI] = 10) of the different *S*. *suis* strains and incubated for 2 h or 4 h at 37 °C in 5% CO_2_. The adhesion assay, which quantifies total cell-associated bacteria (surface-adherent and intracellular bacteria), and invasion assay (using the antibiotic protection assay) were performed as previously described [45].

### J774A.1 macrophage culture and phagocytosis assays

J774A.1 murine macrophages (ATCC TIB-67; Rockville, MD, USA) were maintained in Dulbecco’s Modified Eagle’s Medium (Gibco, Burlington, ON, Canada) supplemented with 10% fetal bovine serum (Gibco) and grown at 37 °C with 5% CO_2_. Confluent cell cultures were scraped, seeded at 1 x 10^5^ cells/mL, and incubated for 3 h at 37 °C with 5% CO_2_ to allow cell adhesion. Cells were infected by adding 1 x 10^7^ CFU/mL of bacterial suspension in complete culture medium (MOI = 100), incubated for 1 h or 2 h at 37 °C with 5% CO_2_, and phagocytosis assays performed as previously described using the antibiotic protection assay [36]. When needed, bacteria were pre-opsonized with 20% complete or heat-inactivated normal mouse serum for 30 min at 37 °C with shaking as previously described [17].

### Murine whole blood bactericidal (killing) assay

Blood was collected from six- to ten-week-old female CD-1 mice (Charles River Laboratories, Wilmington, MA) by exsanguination (800 μL of blood on average) following euthanasia with CO_2_ and mixed with sodium heparin (Sigma-Aldrich). Leukocytes (9 x 10^6^ cells/mL on average) were transferred to a microtube containing 9 x 10^6^ CFU/mL of the different *S*. *suis* strains (MOI = 1) and incubated for 2 h, mixing every 20 min. Assay conditions were chosen based on the kinetics of *S*. *suis* killing by murine blood [36]. After incubation, cells were lysed and appropriate dilutions plated on THA to determine viable bacterial counts. Resistance to bacterial killing by blood leukocytes was compared to incubation in plasma alone (obtained by centrifuging whole blood at 1 800 x *g* for 10 min at 4 °C). The percentage of bacteria killed was determined using the following formula:

1−(bacteriainblood/bacteriainplasma)x100%.

### Porcine whole blood bactericidal (killing) assay

Ten milliliters of blood were collected the jugular vein of four-week-old specific-pathogen free piglets and mixed with sodium heparin (Sigma-Aldrich). The animals originated from a herd free of major important diseases including porcine reproductive, *Mycoplasma hyopneumoniae* and clinical disease related to porcine circovirus. The herd did not have any episode of acute disease related to *S*. *suis*. Leukocytes (9 x 10^6^ cells/mL on average) were transferred to a microtube containing 9 x 10^5^ CFU/mL of the different *S*. *suis* strains (MOI = 0.1) and incubated for 2 h, mixing every 20 min. After incubation, cells were lysed and appropriate dilutions plated on THA to determine viable bacterial counts. Resistance to bacterial killing by blood leukocytes was compared to incubation in plasma alone (obtained by centrifuging whole blood at 1 800 x *g* for 10 min at 4 °C). The percentage of bacteria killed was determined using the following formula:

1−(bacteriainblood/bacteriainplasma)x100%.

### Generation of bone marrow-derived dendritic cells and activation

The femur and tibia from C57BL/6, MyD88^-/-^ (B6.129P2(SJL)-*MyD88*^*tm1*.*Defr*^/J), TLR2^-/-^ (B6.129-*Tlr2*^*tmKir*^/J), and TLR4^-/-^ (B6.B10ScN-*Tlr4*^*lps-del*^/JthJ) mice on C57BL/6J background (Jackson Research Laboratories, Bar Harbor, ME, USA) were used to generate bone marrow-derived DCs, as previously described [17]. Briefly, hematopoietic bone marrow stem cells were cultured in RPMI-1640 medium supplemented with 5% heat-inactivated fetal bovine serum, 10 mM HEPES, 2 mM L-glutamine, and 50 μM 2-mercaptoethanol (Gibco) and complemented with 20% granulocyte-macrophage colony-stimulating factor from mouse-transfected Ag8653 cells [46]. Cell purity was confirmed to be higher than 85% CD11c^+^ by flow cytometry as previously described [17]. Albeit this culture system cannot completely rule out the presence of other innate cells (such as macrophages), it represents an enriched source of DCs. Consequently, cytokines produced by contaminating cells would be minor [47]. Prior to infection, cells were resuspended at 1 x 10^6^ cells/mL in complete medium and stimulated with the different *S*. *suis* strains (1 x 10^6^ CFU/mL; initial MOI = 1). Conditions used were based on those previously published [17, 20]. Supernatants were collected 16 h following infection with *S*. *suis*, time at which secreted cytokine levels were maximal in the absence of *S*. *suis*-induced DC cytotoxicity as determined by lactate dehydrogenase release [17, 20]. Non-infected cells served as negative controls. Secreted levels of tumor necrosis factor (TNF), interleukin (IL)-6, C-C motif chemokine ligand (CCL) 2, CCL3, and C-X-C motif chemokine ligand (CXCL) 1 were quantified by sandwich ELISA using pair-matched antibodies from R&D Systems (Minneapolis, MN, USA) according to the manufacturer’s recommendations.

### *S*. *suis* mouse model of systemic infection

A CD-1 mouse model of infection was used [21, 37]. These studies were carried out in strict accordance with the recommendations of and approved by the Université de Montréal Animal Welfare Committee guidelines and policies, including euthanasia to minimize animal suffering using humane endpoints, applied throughout this study when animals were seriously affected since mortality was not an endpoint measurement. No additional considerations or housing conditions were required. All staff members received the required animal handling training as administered by the Université de Montréal Animal Welfare Committee. Thirty 6-week-old female CD-1 mice (Charles River Laboratories) were used for these experiments (15 mice per strain). Mice were inoculated with 1 x 10^7^ CFU via the intraperitoneal route and health and behavior monitored at least thrice daily until 72 h post-infection and twice thereafter until the end of the experiment (10 days post-infection) for the development of clinical signs of sepsis, such as depression, swollen eyes, rough hair coat, prostration, and lethargy. Clinical scores were determined according to the grid approved by the Université de Montréal Animal Welfare Committee (S3 Appendix) and required actions undertaken. Mice were immediately euthanized upon reaching endpoint criteria using CO_2_ followed by cervical dislocation. No mice died before meeting endpoint criteria and all surviving mice were euthanized as described above at the end of the experiment (10 days post-infection). Blood samples (5 μL) were collected from the caudal vein of surviving mice 12 h and 24 h post-infection and plated as previously described [36].

### Measurement of plasma (systemic) pro-inflammatory mediators

In addition, six mice per group were intraperitoneally mock-infected (THB) or infected with 1 x 10^7^ CFU and blood collected 12 h post-infection by intracardiac puncture (exsanguination; 800 μL of blood on average) following euthanasia with CO_2_ and anti-coagulated with EDTA (Sigma-Aldrich) as previously described [36, 48]. Plasma supernatants were collected following centrifugation at 10 000 x *g* for 10 min at 4 °C and stored at -80 °C. The 12 h post-infection time point was selected to obtain maximal pro-inflammatory mediator production in the absence of significant mouse mortality as determined in a preliminary study (S4 Appendix). Plasmatic concentrations of IL-6, IL-12p70, interferon (IFN)-γ, CCL2, CCL3, CCL4, CCL5, and CXCL2 were measured using a custom-made cytokine Bio-Plex Pro^™^ assay (Bio-Rad, Hercules, CA, USA) according to the manufacturer’s instructions. Mediators were selected based on serotype 2 and serotype 9 studies and represent the most important pro-inflammatory cytokines and chemokines secreted [11, 35–38]. Acquisition was performed on the MAGPIX platform (Luminex^®^) and data analyzed using the Bio-Plex Manager 6.1 software (Bio-Rad).

### Statistical analyses

Normality of data was verified using the Shapiro-Wilk test. Accordingly, parametric (unpaired t-test) or non-parametric tests (Mann-Whitney rank sum test), where appropriate, were performed to evaluate statistical differences between groups. Log-rank (Mantel-Cox) tests were used to compare survival between wild-type-infected mice and those infected with the non-encapsulated strains. Each *in vitro* test was repeated in at least three independent experiments. *p* < 0.05 was considered as statistically significant.

## Results

### Deletion of the *S*. *suis* serotype 9 *cpsG* gene causes a non-encapsulation phenotype

**Fig 1:**
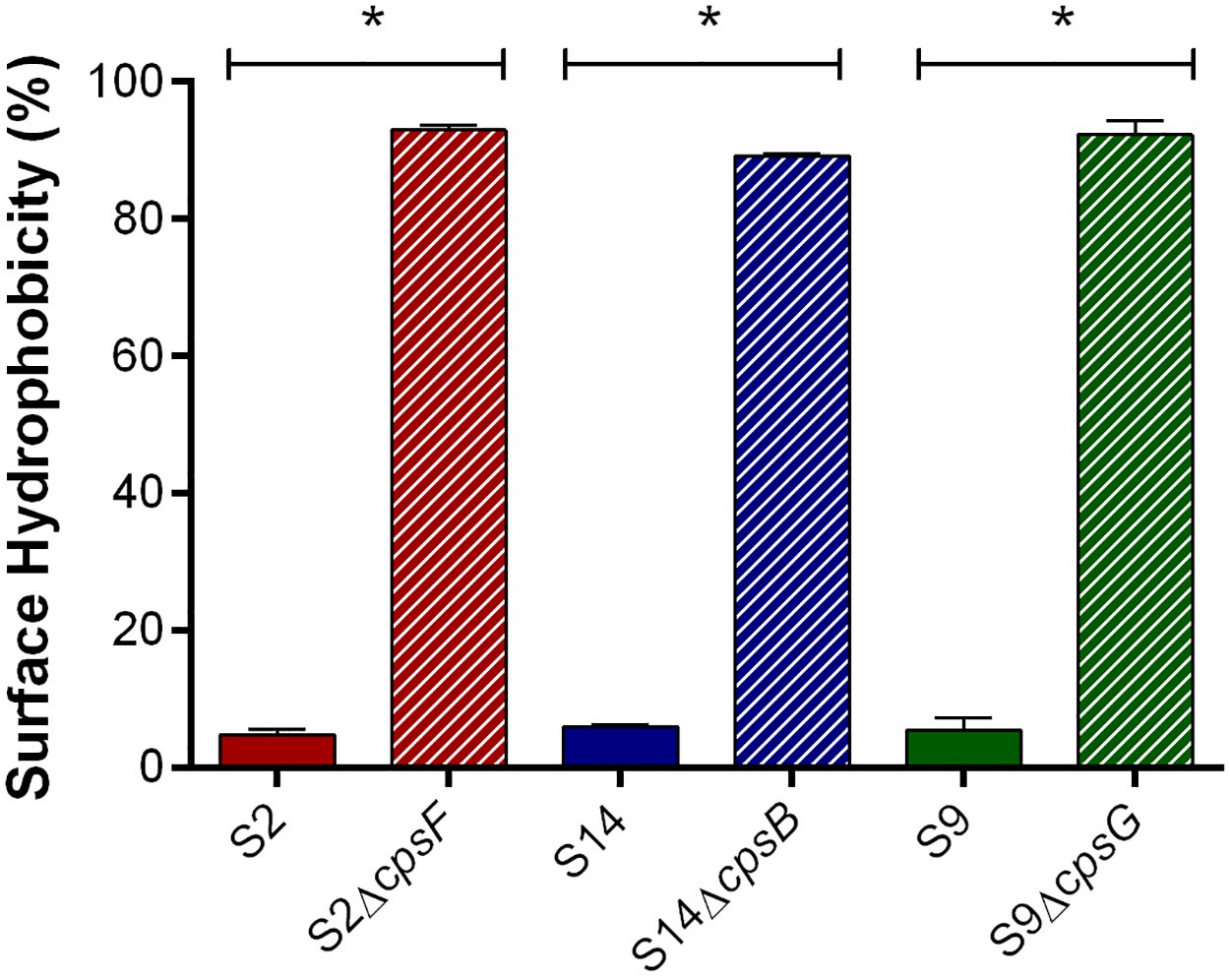
Absence of *S*. *suis* CPS is associated with increased surface hydrophobicity. Surface hydrophobicity of the *S*. *suis* serotype 2 (S2), 9 (S9) or 14 (S14) wild-type and mutant strains was determined using *n*-hexadecane. Data represent the mean ± SEM (n = 3). * (*p* < 0.05) indicates a significant difference between wild-type and mutant strains.

Previous studies have demonstrated that deletion of various CPS biosynthesis genes from serotypes 2 and 14 results in a non-encapsulated phenotype [3, 17, 18, 21, 22, 49]. As such, an isogenic mutant in which the *cpsG* gene, encoding a glycosyltransferase, was deleted from the North American serotype 9 1135776 strain was constructed and compared with serotypes 2 and 14. Surface hydrophobicity (an indicator of encapsulation) of serotype 9 was low (less than 5%) and comparable to that of serotypes 2 and 14 (Fig 1). Meanwhile, deletion of the *cpsG* gene significantly increased surface hydrophobicity (*p* = 0.0002), with similar values to those obtained with the serotype 2 and 14 non-encapsulated mutants (*p* = 0.0002) (Fig 1). This confirms that deletion of these *S*. *suis* CPS biosynthesis genes results in high surface hydrophobicity.

**Fig 2:**
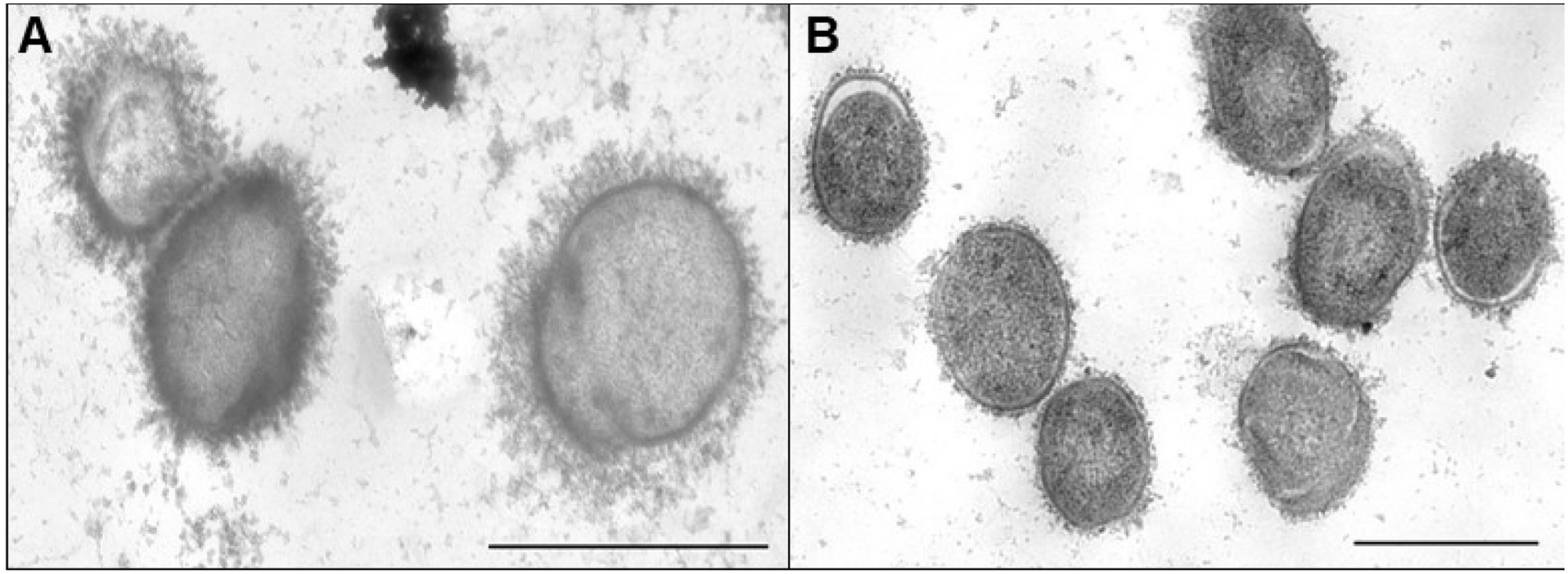
Deletion of *S*. *suis* serotype 9 *cpsG* gene results in non-encapsulation. Transmission electron microscopy following stabilization with ferritin of the serotype 9 wild-type (20 000x magnification) (**A**) and mutant strain (15 000x magnification) (**B**). Black bars = 1 μm.

Encapsulation was then confirmed by transmission electron microscopy following ferritin labelling. The serotype 9 wild-type strain possessed a layer of CPS at its surface, indicative of being well-encapsulated (Fig 2A), that is similar to that of the serotype 2 wild-type P1/7 strain and serotype 14 wild-type DAN13730 strain used herein [3, 21, 35]. Meanwhile, the serotype 9 *cpsG* mutant clearly lacked presence of CPS (Fig 2B), as previously reported for the serotype 2 and 14 non-encapsulated mutants [3, 21]. Consequently, these results confirm that deletion of the *cpsG* gene from serotype 9 results in non-encapsulation.

### Presence of CPS significantly modulates *S*. *suis* serotype 9 invasion of respiratory epithelial cells, but only slightly affects bacterial adhesion to these cells

**Fig 3:**
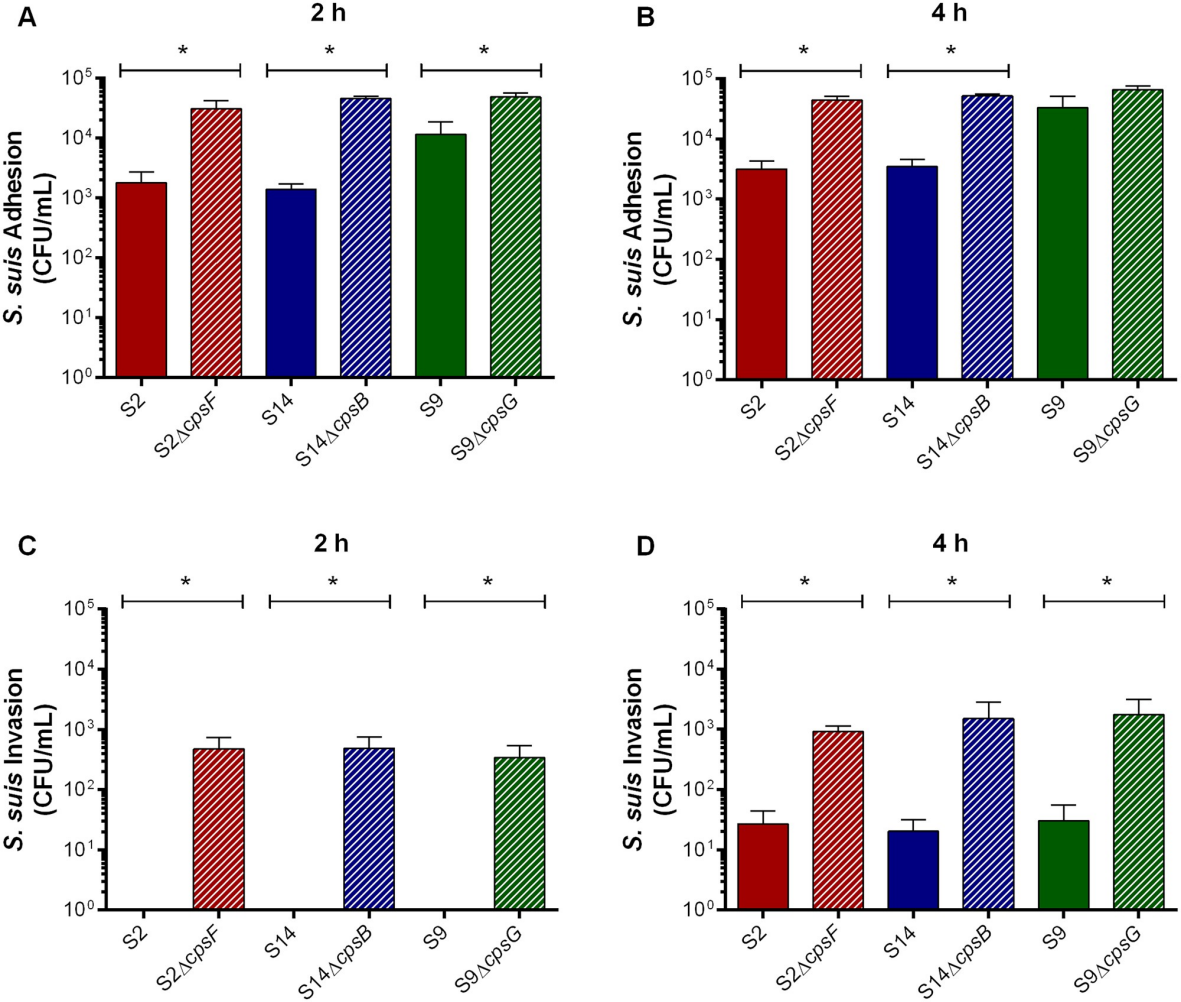
Presence of CPS partially or significantly modulates *S*. *suis* serotype 9 adhesion to and invasion of respiratory epithelial cells, respectively. Adhesion (**A** & **B**) and invasion (**C** & **D**) of the *S*. *suis* serotype 2 (S2), 9 (S9) or 14 (S14) wild-type and mutant strains to NPTr porcine tracheal epithelial cells after 2 h (**A** & **C**) or 4 h (**B** & **D**) of incubation. Data represent the mean ± SEM (n = 4). * (*p* < 0.05) indicates a significant difference between wild-type and mutant strains.

The serotype 2 CPS has been described to mask bacterial surface adhesins involved in the initial interactions with host cells, including adhesion to and invasion of epithelial cells [5]. Using NPTr porcine tracheal epithelial cells, the adhesion and invasion capacities of the different wild-type and non-encapsulated mutant strains were evaluated. The serotype 9 wild-type strain adhered to epithelial cells after 2 h of incubation, with bacterial adhesion increasing by 4 h (Fig 3A and 3B). However, adhesion of serotype 9 was significantly greater than that of serotypes 2 and 14 regardless of incubation time (*p* = 0.012) (Fig 3A and 3B). Meanwhile, although non-encapsulation significantly increased adhesion of serotypes 2 and 14 regardless of incubation time (*p* = 0.002), that of serotype 9 slightly modulated the adhesion to epithelial cells, but only at 2 h of incubation (*p* = 0.027) (Fig 3A and 3B). Consequently, these results suggest that presence of the serotype 9 CPS masks surface adhesins less efficiently.

Following adhesion, *S*. *suis* may invade cells. However, invasion of epithelial cells by serotype 9 was extremely low (less than 20 CFU/mL), even after 4 h of incubation, which was similar to that by serotypes 2 and 14 (Fig 3C and 3D), indicating that higher adhesion is not necessarily followed by increased bacterial internalization. On the other hand, invasion by the non-encapsulated serotype 9 mutant was significantly greater at both 2 h and 4 h than the wild-type strain (*p* = 0.008) (Fig 3C and 3D). Similar results were obtained for the non-encapsulated serotype 2 and 14 mutants (*p* = 0.008), and levels of internalized bacteria were comparable between the three serotypes (Fig 3C and 3D).

### Presence of CPS confers resistance to phagocytosis by macrophages independently of the *S*. *suis* serotype

**Fig 4:**
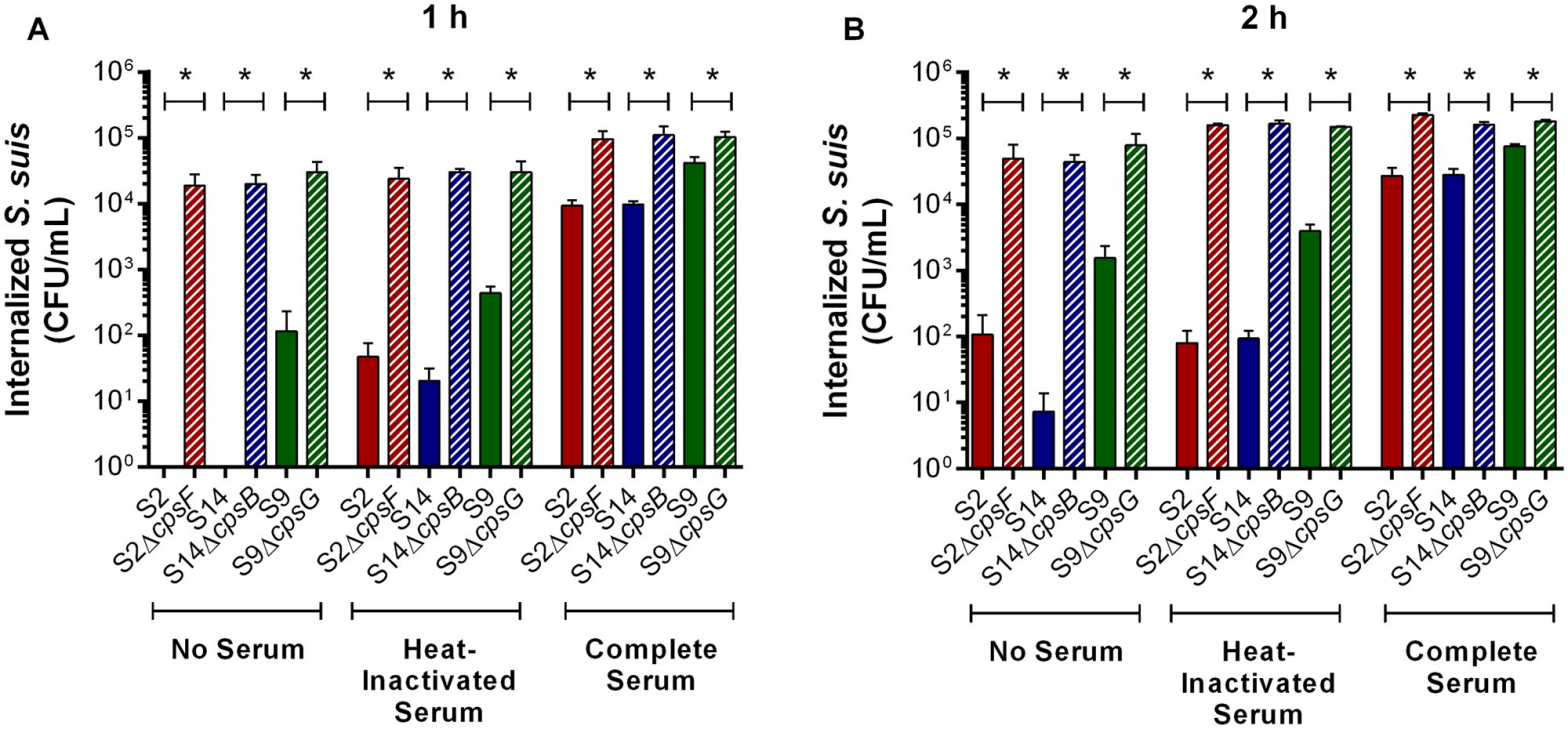
Presence of *S*. *suis* CPS confers anti-phagocytic properties towards macrophages regardless of serotype. Internalization of the *S*. *suis* serotype 2 (S2), 9 (S9) or 14 (S14) wild-type and mutant strains by J774A.1 murine macrophages after 1 h (**A**) or 2 h (**B**) of incubation in the absence of mouse serum or following opsonization with 20% heat-inactivated or complete serum. Data represent the mean ± SEM (n = 4). * (*p* < 0.05) indicates a significant difference between wild-type and mutant strains.

Phagocytosis studies with *S*. *suis* serotype 9 were carried out with J774A.1 murine macrophages, as previously done with serotypes 2 and 14 [3, 16, 36]. Serotype 9 was already internalized after 1 h of incubation, and this even in the absence of serum, with intracellular bacteria increasing over time (Fig 4A and 4B). By contrast, serotypes 2 and 14 were not internalized (1 h) or significantly less internalized (2 h) by macrophages than serotype 9 (*p* = 0.008) (Fig 4A and 4B). Moreover, similar patterns were also obtained using complete and heat-inactivated mouse sera, though levels of internalized *S*. *suis* were greatest in the presence of complete serum, and this regardless of serotype (Fig 4A and 4B).

Of the different properties attributed to the presence of the *S*. *suis* serotype 2 and 14 CPSs, resistance to phagocytosis is amongst the most important [3, 16, 21]. Indeed, results were confirmed in the present study (*p* = 0.002) (Fig 4A and 4B). Results also showed that non-encapsulation of serotype 9 significantly increased phagocytosis by macrophages (*p* = 0.01), with levels of intracellular bacteria remaining stable over time (Fig 4A and 4B). However, internalization of the non-encapsulated mutant was similar when using complete or heat-inactivated serum (Fig 4A and 4B). Taken together, these results suggest that though the *S*. *suis* CPS confers anti-phagocytic properties regardless of the serotype, the serotype 9 CPS protects bacteria less efficiently than those of serotypes 2 and 14.

### Presence of CPS is required for *S*. *suis* whole blood bactericidal resistance regardless of serotype

**Fig 5:**
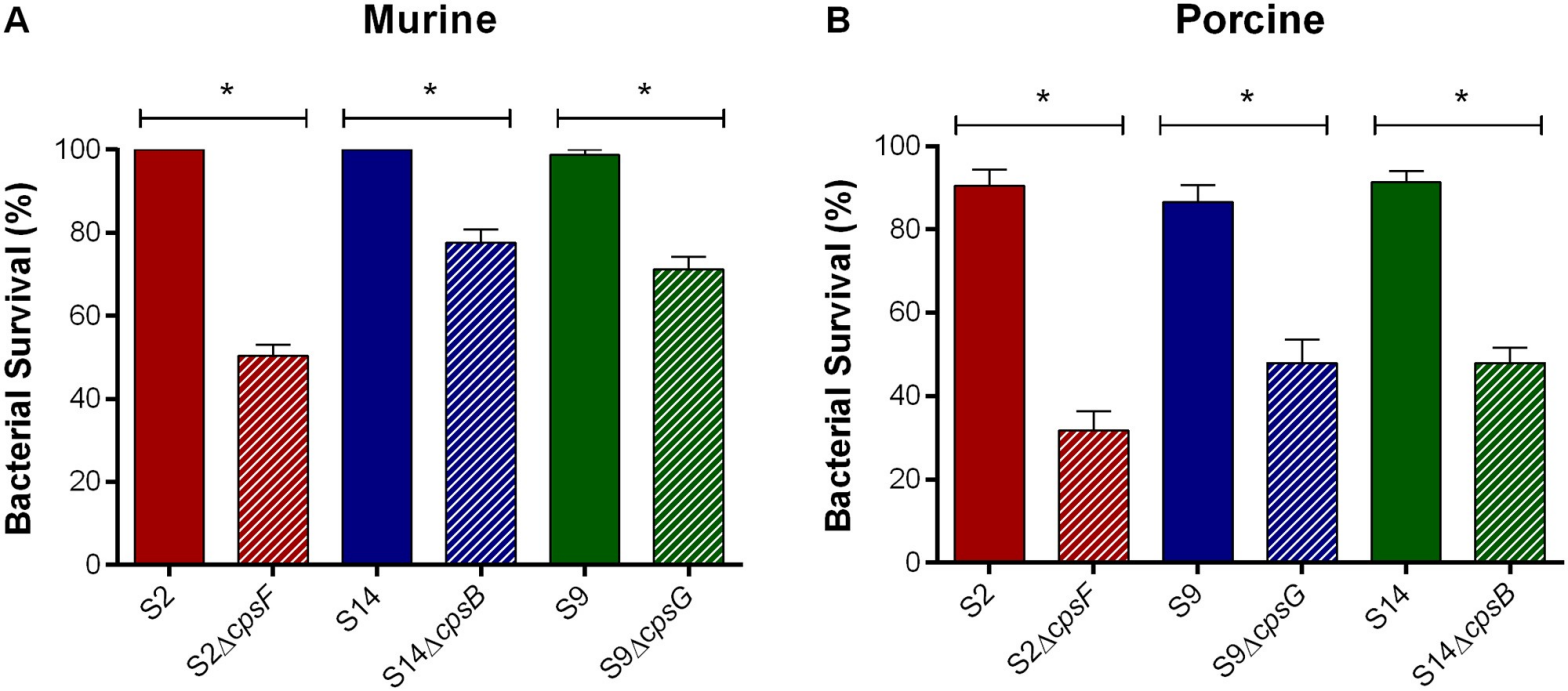
Presence of CPS is required for *S*. *suis* whole blood bactericidal resistance regardless of serotype. Capacity of the *S*. *suis* serotype 2 (S2), 9 (S9) or 14 (S14) wild-type and mutant strains to resist the bactericidal effect of murine (**A**) or porcine (**B**) whole blood after 2 h of incubation. Percentage of bacterial survival was calculated in comparison to bacteria in plasma alone. Data represent the mean ± SEM (n = 3). * (*p* < 0.05) indicates a significant difference between wild-type and mutant strains.

Results showed that the serotype 9 wild-type strain was as resistant to murine whole blood bacterial killing as serotypes 2 and 14 (Fig 5A). Confirming previous data [3, 18, 21, 36], results also showed that the CPS is required for survival and persistence of *S*. *suis* serotypes 2 and 14 in murine blood (Fig 5A). In addition, the serotype 9 non-encapsulated mutant was significantly less resistant to killing (*p* = 0.0002) than its wild-type strain, with 30% of bacteria killed after 2 h (Fig 5A). Although the serotype 9 and 14 non-encapsulated mutants were comparably killed, they were both significantly more resistant than the serotype 2 non-encapsulated mutant (*p* = 0.02) (Fig 5A).

Results obtained using murine whole blood were then confirmed using porcine whole blood, with the serotype 9 wild-type strain being as resistant to bacterial killing as serotypes 2 and 14 and the presence of CPS significantly promoting survival of all three serotypes (*p* = 0.0001) (Fig 5B). Moreover, the serotype 9 and 14 non-encapsulated mutants were also comparably killed, yet significantly more resistant than the serotype 2 non-encapsulated mutant (*p* = 0.04) (Fig 5B).

### Modulation of dendritic cell pro-inflammatory mediator production by presence of *S*. *suis* CPS and recognition by the Toll-like receptor pathway are comparable between serotypes

**Fig 6:**
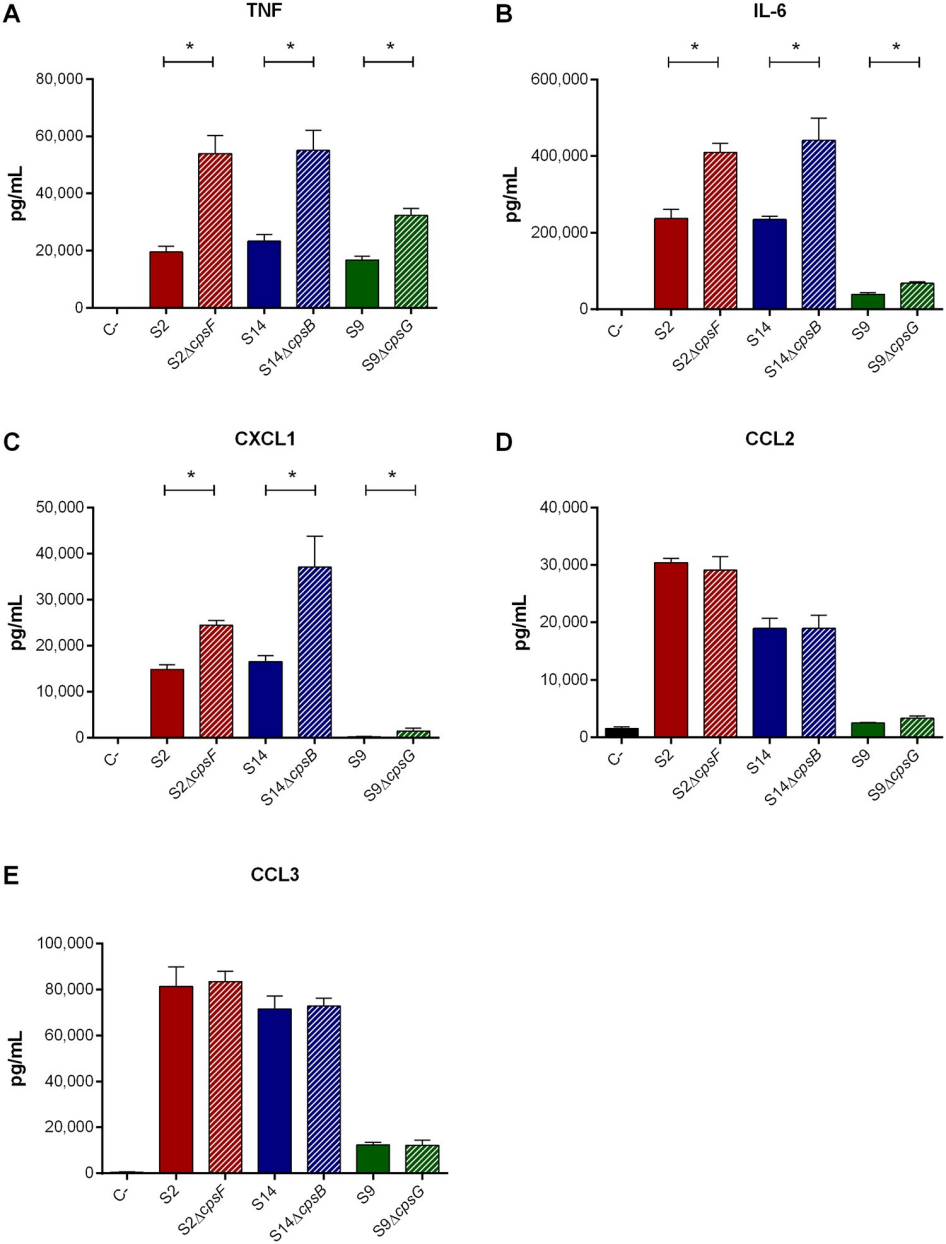
Presence of CPS modulates *S*. *suis*-induced pro-inflammatory mediator production by dendritic cells (DCs) regardless of serotype. Pro-inflammatory mediator production by DCs following infection with the *S*. *suis* serotype 2 (S2), 9 (S9) or 14 (S14) wild-type and mutant strains after 16 h of incubation, as measured by ELISA. Production of TNF (**A**), IL-6 (**B**), CXCL1 (**C**), CCL2 (**D**), and CCL3 (**E**). Data represent the mean ± SEM (n = 4). C- denotes cells in medium alone. * (*p* < 0.05) indicates a significant difference between wild-type and mutant strains.

Bone marrow-derived DCs were used as an innate immune cell model given that DCs play a critical role during *S*. *suis* pathogenesis and that their response to *S*. *suis* serotype 2 has been well-characterized [17, 18, 20, 35, 50]. Surprisingly, and except for TNF, the serotype 9 strain induced lower levels of the different pro-inflammatory mediators evaluated, especially of CCL2 and CXCL1, when compared to serotypes 2 and 14 (*p* = 0.006) (Fig 6). Meanwhile, levels induced by serotypes 2 and 14 were comparable (Fig 6).

Alongside phagocytosis resistance, the serotype 2 CPS has been well-described to interfere with recognition of *S*. *suis* by innate immune cells by masking immunostimulatory bacterial surface components [5], a property not yet investigated for serotypes 9 and 14. Lack of CPS significantly increased production of TNF, IL-6, and CXCL1 (*p* = 0.01), but not of CCL2 nor CCL3 by serotype 9, with similar results for serotypes 2 and 14 (*p* = 0.001) (Fig 6). Consequently, the inflammatory response induced by serotype 9 from DCs is markedly lower than that of serotypes 2 and 14, with presence of its CPS modulating this production somewhat less.

**Fig 7:**
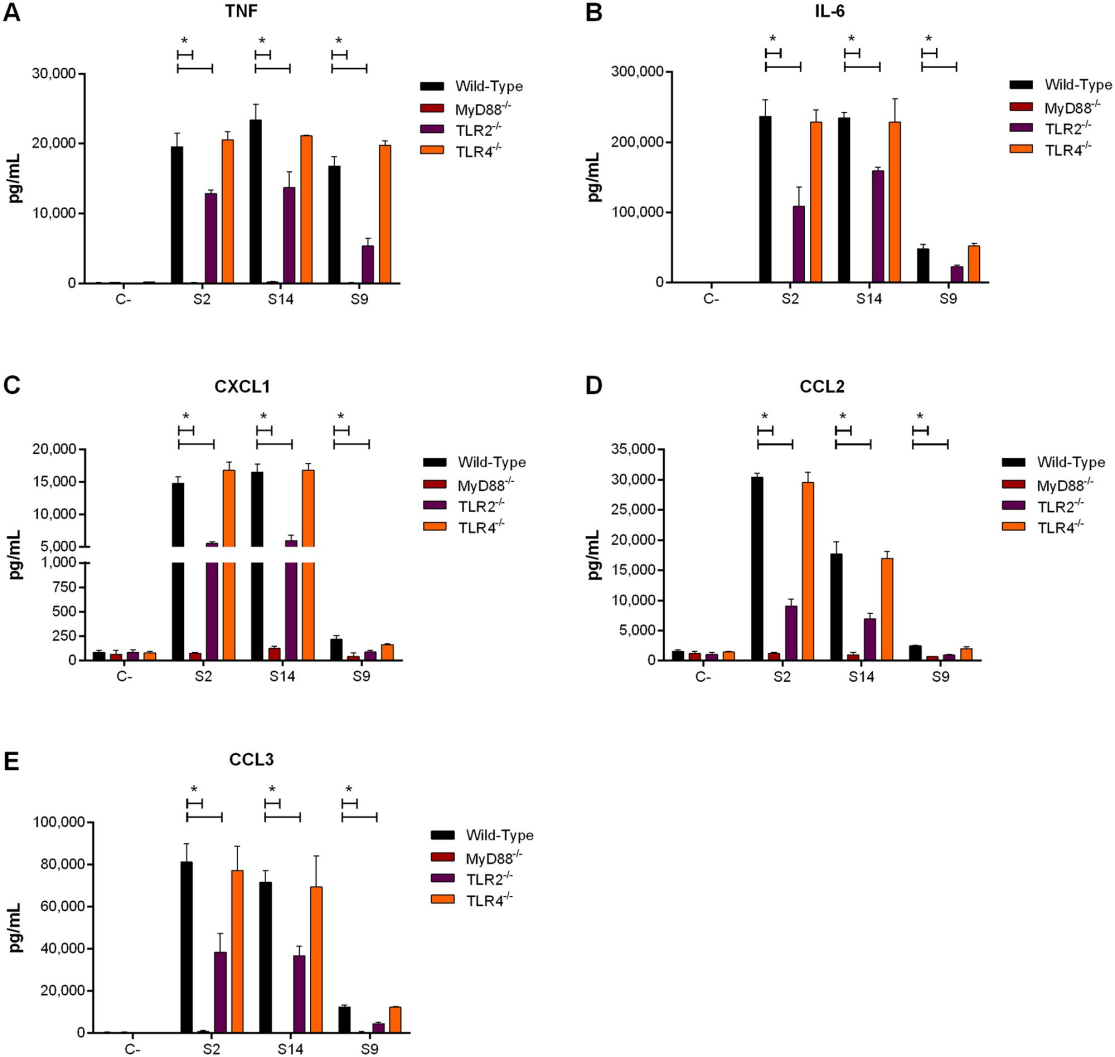
MyD88-dependent Toll-like receptor (TLR) signaling is required for *S*. *suis*-induced pro-inflammatory mediator production by dendritic cells (DCs) regardless of serotype. Pro-inflammatory mediator production by wild-type, MyD88^-/-^, TLR2^-/-^, and TLR4^-/-^ DCs following infection with the *S*. *suis* serotype 2 (S2), 9 (S9) or 14 (S14) wild-type strains after 16 h of incubation, as measured by ELISA. Production of TNF (**A**), IL-6 (**B**), CXCL1 (**C**), CCL2 (**D**), and CCL3 (**E**). Data represent the mean ± SEM (n = 4). C- denotes cells in medium alone. * (*p* < 0.05) indicates a significant difference between wild-type and knockout cells.

*S*. *suis*-induced inflammation results from its recognition by host cells via different pathways, of which the TLR pathway has been best described for serotype 2 [5]. Moreover, since recognition of *S*. *suis* serotype 2 mainly occurs at the host cell surface, TLR2 is the main receptor involved [19, 20]. In addition, TLR4 has been suggested to recognize the pore-forming toxin suilysin [51], produced by the serotype 2 and 14 strains used herein, but not by the serotype 9 strain [34, 52]. Importantly, recruitment of the adaptor protein MyD88 is central to the TLR pathway [53]. As such, the role of the TLR pathway in the recognition of *S*. *suis* by DCs was investigated. Absence of MyD88 led to a near complete abrogation of pro-inflammatory mediator production induced by serotype 9, similarly to serotypes 2 and 14 (*p* = 0.0002) (Fig 7). Moreover, production induced by serotype 9 was partially dependent on TLR2, with a 50% to 60% decrease in its absence (*p* = 0.02), which was also similar to what was observed with serotypes 2 and 14 (*p* = 0.01) (Fig 7). By contrast, recognition of serotype 9, as with serotypes 2 and 14, was TLR4-independent (Fig 7). Taken together, these results demonstrate that pro-inflammatory mediator production induced by *S*. *suis* serotype 9 from DCs is MyD88-dependent and partially requires TLR2 but not TLR4.

### Presence of CPS is required for virulence of *S*. *suis* serotype 9 and development of systemic disease in a mouse model of infection

**Fig 8:**
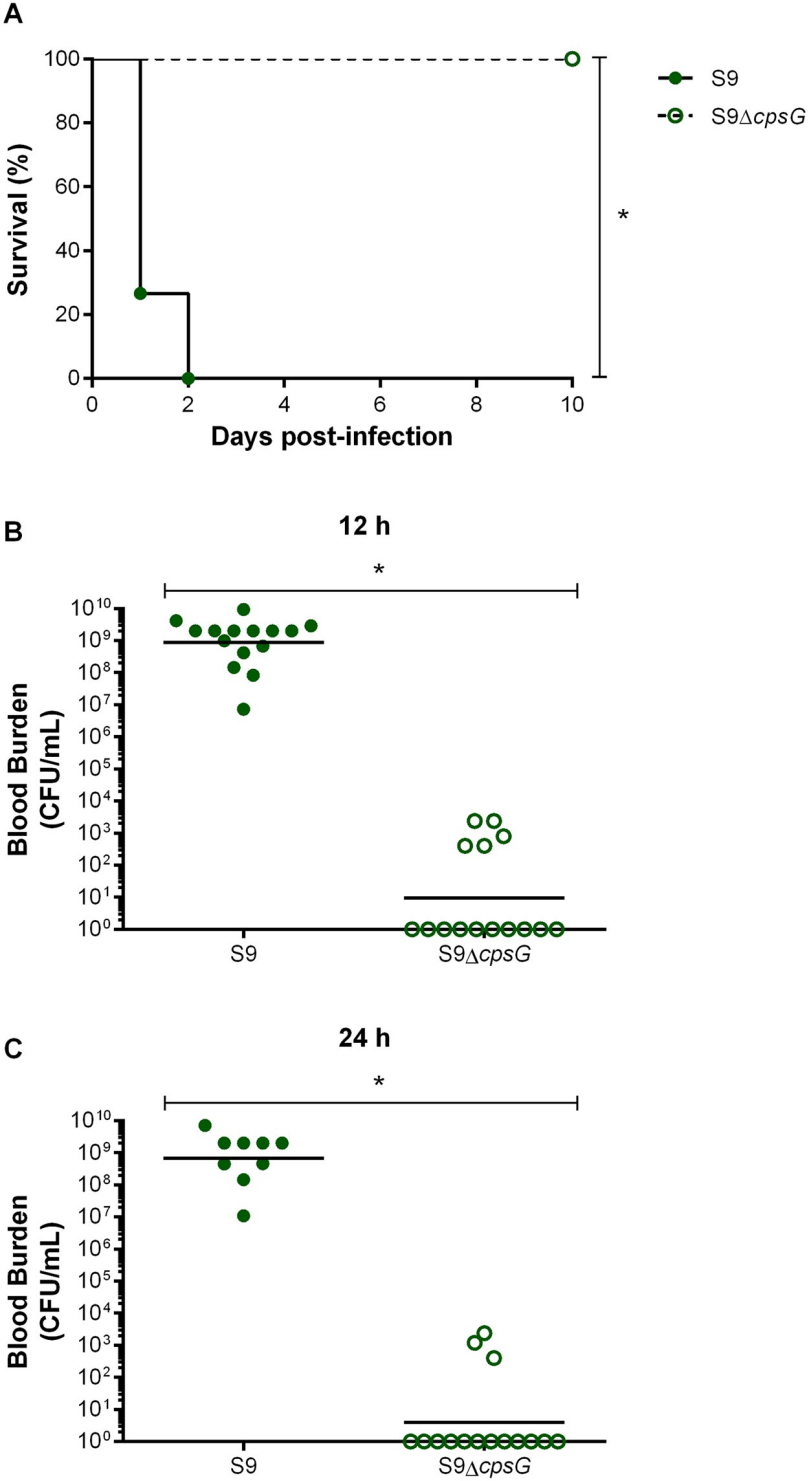
CPS is required for *S*. *suis* serotype 9 virulence and persistence in blood in a mouse model of infection. Survival (**A**) and blood bacterial burden 12 h (**B**) and 24 h post-infection (**C**) of CD-1 mice following intraperitoneal inoculation of the *S*. *suis* serotype 9 wild-type (S9) or mutant (S9Δ*cpsG*) strain. Data represent survival curves (A) or geometric mean (B & C) (n = 15). * (*p* < 0.05) indicates a significant difference between survival or blood bacterial burden of mice infected with wild-type and mutant strain.

To evaluate the role of the *S*. *suis* serotype 9 CPS in virulence and development of clinical disease, CD-1 mice, commonly used for serotype 2 virulence studies [18, 21, 37], were infected with the serotype 9 wild-type and mutant strain by intraperitoneal inoculation. Wild-type strain-infected mice rapidly developed clinical signs of systemic disease characteristic of septic shock with 100% of mice succumbing to infection within 48 h (Fig 8A). By contrast, none of the mice infected with the non-encapsulated mutant succumbed to disease (*p* = 0.0002) (Fig 8A). In fact, these mice only developed transient signs of infection such as rough coat hair following inoculation of bacteria (S5 Appendix). Viability of the inoculum was verified prior to and after infection with no differences between.

To better explain differences in virulence, and since *S*. *suis* systemic infection is associated with persistent bacteremia [11, 36], blood bacterial burden was evaluated 12 h and 24 h post-infection. All mice infected with the wild-type strain presented elevated blood bacterial burdens that averaged 5 x 10^8^ CFU/mL (Fig 8B and 8C). In fact, levels were comparable to those obtained upon euthanasia of mice suffering from septic shock (2 x 10^9^ CFU/mL). On the other hand, almost no bacterial burden was detected in the blood of mice infected with the non-encapsulated mutant, levels of which were not only significantly lower than those of mice infected with the wild-type strain (*p* = 0.0002), but almost undetectable (Fig 8B and 8C).

**Fig 9:**
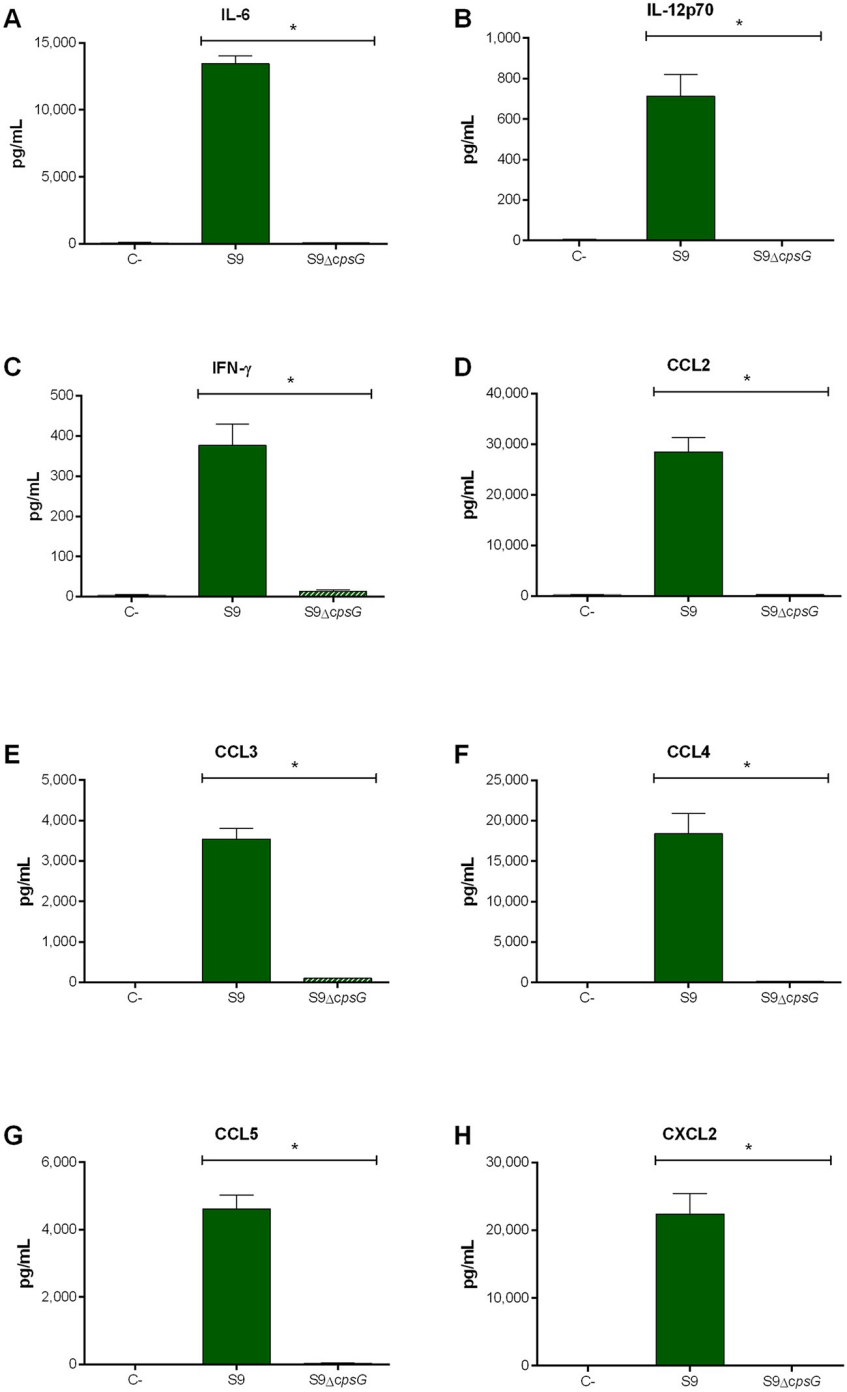
Reduced plasma pro-inflammatory mediator levels in the absence of *S*. *suis* serotype 9 CPS during systemic infection. Plasma levels of IL-6 (**A**), IL-12p70 (**B**), IFN-γ (**C**), CCL2 (**D**), CCL3 (**E**), CCL4 (**F**), CCL5 (**G**), and CXCL2 (**H**) in mice 12 h following following mock-infection or intraperitoneal inoculation of the *S*. *suis* serotype 9 wild-type (S9) or mutant (S9Δ*cspG*) strain. Data represent mean ± SEM (n = 6). C- denotes mock-infected mice. * (*p* < 0.05) indicates a significant difference between plasma levels of mice infected with the wild-type strain and mutant strain.

Furthermore, exacerbated inflammation is a hallmark of the *S*. *suis*-induced systemic infection and is responsible for host death due to septic shock [11, 36]. In accordance, plasmatic levels of the different pro-inflammatory mediators (IL-6, IL-12p70, IFN-γ, CCL2, CCL3, CCL4, CCL5, and CXCL2) were elevated in mice infected with the serotype 9 wild-type strain (Fig 9). In contrast, levels of these mediators were significantly lower in mice infected with the non-encapsulated mutant (*p* < 0.001) and were similar to those of mock-infected mice (Fig 9).

## Discussion

Colonization is amongst the first steps of the *S*. *suis* pathogenesis, with CPS expression playing an important role therein for serotype 2 [54–56]. Adhesion of serotype 9 to tracheal epithelial cells was greater than that of serotypes 2 and 14 and, differently from the latter two, its CPS hardly inhibited these interactions. Moreover, it was previously reported that serotype 9 also adhered more to porcine intestinal epithelial cells than did serotype 2 [10]. However, these differences are probably not due to a lower expression or thickness of CPS in serotype 9 since the strains used herein are similarly encapsulated [21, 57]. In fact, yields of purified CPS from serotype 9 are greater than those from serotypes 2 and 14 [25, 26, 33]. Consequently, these results might suggest that the serotype 9 surface adhesins may remain at least partially exposed in the presence of its CPS and/or that serotype 9 may not possess the same adhesins as serotypes 2 and 14 or that their expression levels may differ. Indeed, using these serotype 2 and 9 strains, it was demonstrated that serotype 9 adheres more to extracellular matrix components (collagen I, fibrinogen and fibronectin) and salivary agglutinin proteins than serotype 2, partially due to an important role of the AgI/II for serotype 9 [12].

Though encapsulation inhibited serotype 9 invasion of tracheal epithelial cells by rendering the bacterial factors involved inaccessible, this effect was comparable to serotypes 2 and 14. This indicates that the factors involved in serotype 9 adhesion and invasion probably differ. Interestingly, invasion of intestinal epithelial cells was greater by non-encapsulated serotype 2 than serotype 9 [10], suggesting that different adhesins may be involved in bacterial-host interactions in both the respiratory and intestinal tracts.

It has been reported that the CPS is a critical anti-phagocytic and virulence factor for *S*. *suis* [5, 58]. However, these conclusions are based on serotype 2 and, more recently, on serotype 14 studies [3]. Herein, serotype 9 was more susceptible to phagocytosis than serotypes 2 and 14, with similar results for the latter two. It has been previously demonstrated that serotype 9 is also more internalized by human monocyte-derived DCs [59]. Despite these observations, the absence of CPS significantly increased phagocytosis levels of *S*. *suis* serotype 9, confirming a certain anti-phagocytic role as is the case for serotypes 2 and 14. It can be hypothesized that the composition of the serotypes 2 and 14 CPSs, which are similar, including presence of a sialic acid sidechain, may play a role in such increased resistance to phagocytosis [25, 26, 60, 61]. Indeed, the composition and structure of the CPS of serotype 9 greatly differs from these two serotypes, as it contains glucitol, phosphate, and a labile 4-keto sugar, all of which are absent from the serotype 2 and 14 CPSs, but does not contain sialic acid [33]. Moreover, results demonstrated that complement (evaluated using heat-inactivated and complete mouse sera) was the serum component majorly responsible for increasing sensitivity of serotypes 2, 9, and 14 to phagocytosis. Finally, and similarly to what may happen during the interactions with epithelial cells, the serotype 9 CPS might not mask the surface components responsible for activation of phagocytic mechanisms as efficiently as serotypes 2 and 14.

Although clear differences were observed with the phagocytosis assay, encapsulated serotypes 2, 9 and 14 were all equally resistant to killing by murine and porcine whole blood, with the CPS playing a significant role in blood survival. An important difference is that neutrophils and monocytes are the main phagocytes in blood, with little to no macrophages being present [62]. In this study, the protective role of CPS was confirmed *in vivo* using a mouse model, since non-encapsulation of serotype 9 resulted in near complete elimination from the bloodstream after 24 h of infection. In fact, in absence of its CPS, serotype 9 was unable to persist, disseminate systemically, and cause disease, being avirulent. This suggests that the chemical composition and structure of the CPS, including the presence or absence of sialic acid does not influence its capacity to protect *S*. *suis* from killing by leukocytes, at least for the serotypes studied. It cannot be completely excluded that the phenotypes observed were not influenced, at least partially, by the fact that different genes were deleted from serotypes 2, 9 and 14. However, data obtained with serotype 2 indicate that the CPS operon and synthesis is tightly controlled and regulated and that absence of a single gene results in non-encapsulation, and this regardless of strain background [17, 18, 21, 22, 31, 49, 63].

Interestingly, levels of *S*. *suis* killing by porcine whole blood were somewhat greater than those by murine whole blood, probably due to the cross-reacting antibodies present, since 100% of pigs are naturally colonized by *S*. *suis*, including by different serotypes simultaneously [64]. The similarities in the results obtained using murine and porcine whole blood support previous results demonstrating conserved interactions and recognition mechanisms between murine and porcine cells and *S*. *suis*, including by dendritic cells [17, 20, 65]. Similarities between porcine and murine cells may extend to other cell types and functions, including macrophages and phagocytosis, though future studies will be necessary to validate this correlation.

Levels of pro-inflammatory mediators induced by serotype 9 from DCs were, except for TNF, markedly lower than those induced by serotypes 2 and 14, with absence of CPS from serotype 9 also increasing the release of pro-inflammatory mediators. However, this effect was less notable than for serotypes 2 and 14, suggesting that even when present, it only partially masks the lipoproteins and other immunostimulatory surface components involved in DC activation. These results were somewhat unexpected since serotype 9 lipoproteins were suggested to possess greater immunostimulatory properties than those of serotype 2 [59, 66]. Moreover, this lower activation of DCs by serotype 9, even though presence of its CPS does not efficiently mask its adhesins, suggests that adhesins are probably not responsible for the induction of inflammatory mediators. These differences might be due to the cell type used, the use of a single cell type, or the host cell origin. Though serotype 9 induced lower pro-inflammatory mediator production from DCs, its immunostimulatory potential remains notable since 100% of mice infected with the wild-type strain developed septic shock within 48 h of infection, with elevated systemic inflammatory markers already observed 12 h post-infection. This does not seem to be specific to the mouse strain used, since similarly high systemic inflammation was also observed in C57BL/6 mice following infection with the same strain [11]. Interestingly, the levels of plasma mediators induced by serotype 9 are generally comparable to those induced by the serotype 2 31533 strain in CD-1 mice and the serotype 2 P1/7 strain, used herein, in C57BL/6 mice, while no results are available for serotype 14 [36, 37]. The comparable systemic inflammation following serotype 2 and 9 infections may explain the similar clinical signs of infection (due to inflammation) and host death. Moreover, the results obtained *in vivo* indicate that other cell types also contribute to serotype 9-induced systemic inflammation, possibly including monocytes, neutrophils and Natural Killer cells, which are important sources of plasma pro-inflammatory mediators during bacterial infection [67]. While the non-encapsulated mutant induced greater levels of pro-inflammatory mediators from DCs *in vitro*, it induced very little release of plasma mediators in infected mice. This difference can be explained by the lack of resistance of non-encapsulated mutant to the bactericidal effect of whole blood as observed using the killing assay and bacteremia *in vivo*, resulting in its rapid clearance from the systemic compartment and an overall reduced inflammatory activation of host cells *in vivo* [18]. Finally, although differences in inflammatory mediator production were observed, the receptors involved in recognition of serotypes 2, 9, and 14 are similar. Indeed, the importance of the MyD88-dependent TLR pathway was comparable for the three serotypes.

## Conclusions

In conclusion, the interactions between *S*. *suis* serotype 9 and host cells are similar to those of serotypes 2 and 14. Indeed, the serotype 9 CPS is a critical virulence factor required for bacterial survival in blood and development of clinical disease regardless of its unique composition and structure, including absence of sialic acid. Though serotype 9 induces lower production of pro-inflammatory mediators from DCs, it causes an exacerbated inflammatory response, which combined with the persistent bacterial presence, is probably responsible for host death during its systemic infection *in vivo*, suggesting a role of other innate immune cells. Furthermore, recognition of *S*. *suis* requires MyD88-dependent signaling and mostly TLR2, regardless of the serotype, indicating that evolutionarily conserved bacterial components are responsible for initial host cell recognition. However, even when present, the serotype 9 CPS does not mask surface adhesins, nor does it prevent phagocytosis as efficiently as the CPS of serotype 2 or 14.

## Supporting information

S1 Appendix Gel electrophoresis of gene *cpsG* in the *S*. *suis* serotype 9 wild-type strain (S9) and its non-encapsulated mutant (Δ*cpsG*) following amplification by polymerase chain reaction with primers *cpsG*-5 and *cpsG*-8 (see Table 2). Lane 1 = S9; lane 2 = S9Δ*cpsG*; lane 3 = non-template control (C-); lane 4 = molecular weight (MW) ladder. The band corresponding to S9 is 3900 bp and that to *S9*Δ*cpsG* is 3200 bp.
(TIF)

S2 Appendix Growth curves of the *S*. *suis* serotype 9 wild-type strain (S9) and its non-encapsulated mutant (S9Δ*cpsG*). Data represent the mean ± SEM (n = 3).
(TIF)

S3 Appendix Evaluation of clinical signs and scoring following intraperitoneal injection of *Streptococcus suis* in mice. (PDF)

S4 Appendix Plasma pro-inflammatory mediator production kinetic following *S*. *suis* serotype 9 infection. Plasma levels of IL-6 (**A**), IL-12p70 (**B**), CCL2 (**C**), and CXCL2 (**D**) in mice 3 h, 6 h, 12 h, and 24 h following following intraperitoneal inoculation of the *S*. *suis* serotype 9 wild-type strain. Data represent mean ± SEM (n = 3).
(TIF)

S5 Appendix Clinical signs observed in *S*. *suis* serotype 9 wild-type- and non-encapsulated mutant (S9Δ*cpsG*)-infected CD-1 mice following intraperitoneal inoculation (n = 15). (PDF)
