# Supplementary figures and images for: Interactions of Streptococcus suis serotype 9 with host cells and role of the capsular polysaccharide: Comparison with serotypes 2 and 14
Source: PLoS One. 2019 Oct 10;14(10):e0223864. doi: 10.1371/journal.pone.0223864 (PMC6786723; doi:10.1371/journal.pone.0223864)

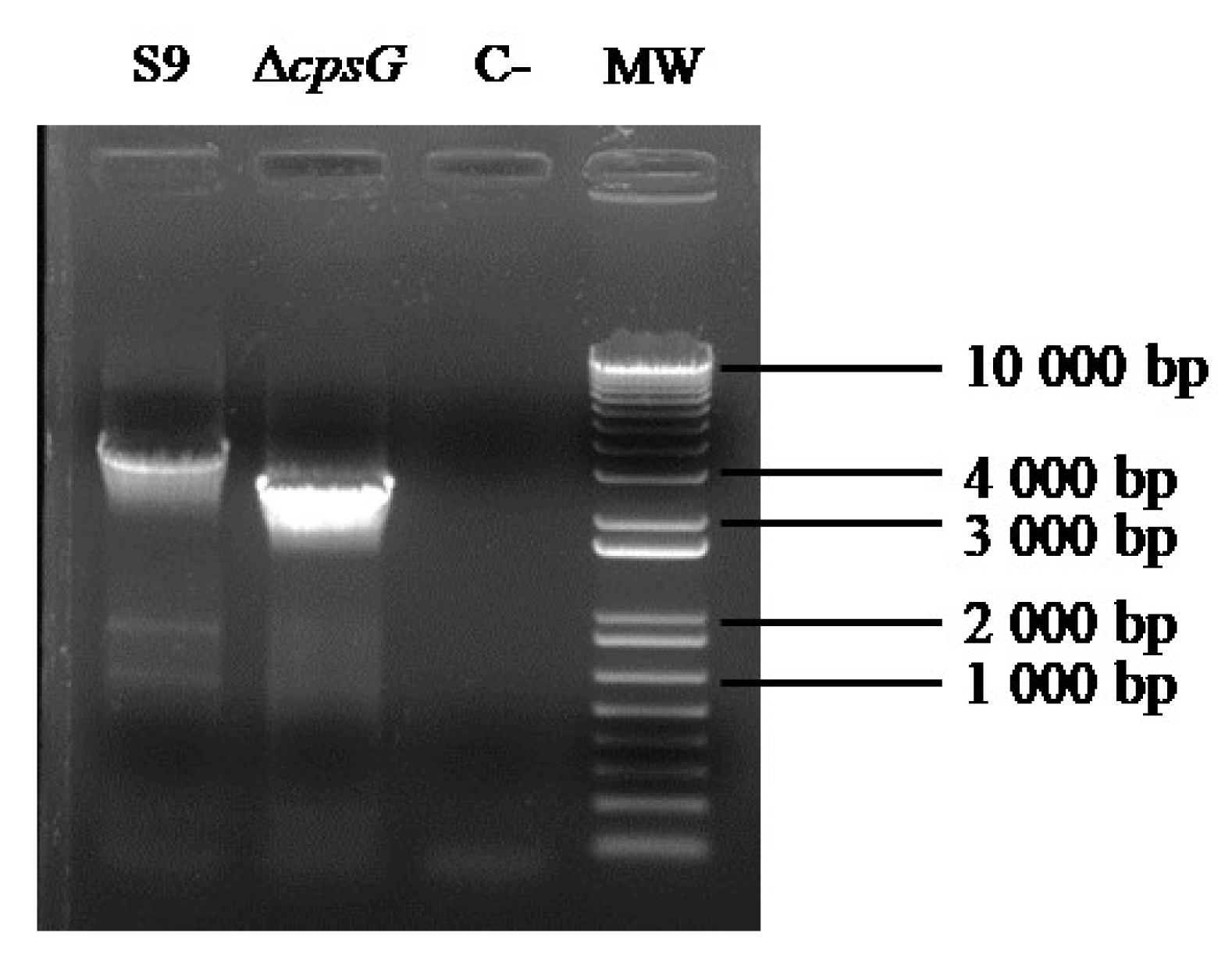

Supplement: S1 Appendix — Lane 1 = S9; lane 2 = S9ΔcpsG; lane 3 = non-template control (C-); lane 4 = molecular weight (MW) ladder. The band corresponding to S9 is 3900 bp and that to S9ΔcpsG is 3200 bp. (TIF) [file pone.0223864.s001.tif]

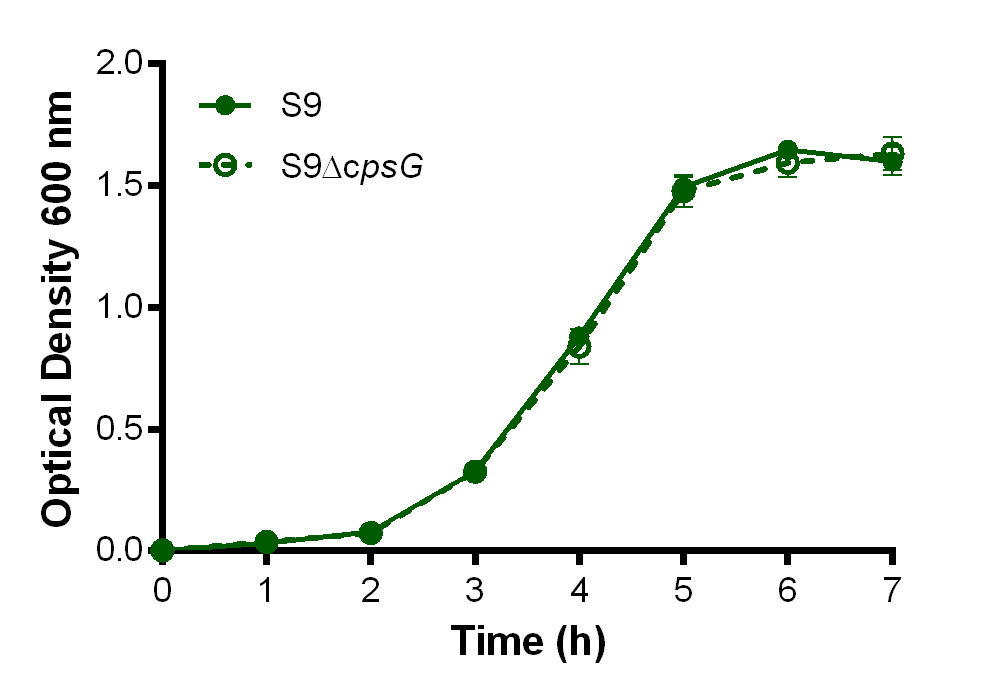

Supplement: S2 Appendix — Data represent the mean ± SEM (n = 3). (TIF) [file pone.0223864.s002.tif]

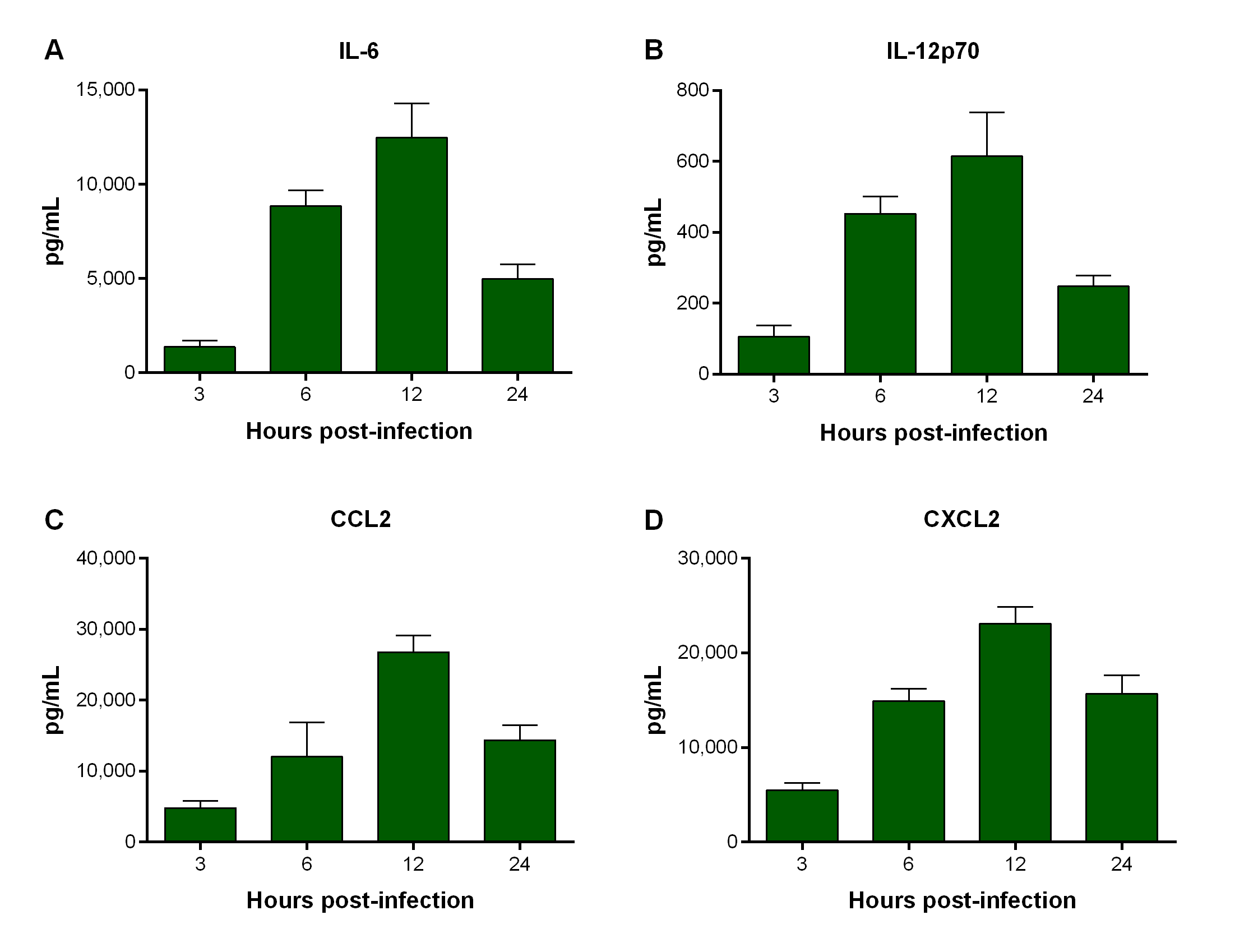

Supplement: S4 Appendix — Plasma levels of IL-6 (A), IL-12p70 (B), CCL2 (C), and CXCL2 (D) in mice 3 h, 6 h, 12 h, and 24 h following following intraperitoneal inoculation of the S. suis serotype 9 wild-type strain. Data represent mean ± SEM (n = 3). (TIF) [file pone.0223864.s004.tif]
