# Supplementary material for: Interactions of Streptococcus suis serotype 9 with host cells and role of the capsular polysaccharide: Comparison with serotypes 2 and 14
Source: PLoS One. 2019 Oct 10;14(10):e0223864. doi: 10.1371/journal.pone.0223864 (PMC6786723; doi:10.1371/journal.pone.0223864)
Supplement: S5 Appendix — (PDF) [file pone.0223864.s005.pdf]

Supporting Information – S5 Appendix

<u>Clinical signs observed in *Streptococcus suis* serotype 9 wild-type- and non-encapsulated mutant (S9Δ*cpsG*)-infected CD-1 mice following intraperitoneal inoculation (n = 15)</u>

<u>Scoring:</u>
0 = normal
> 3 = (transitory) post-infection reaction
4-6 = moderately/visibly sick
< 7 = immediate euthanasia

**Days post-infection**	**<u>Wild-Type (S9)</u>**					**<u>Non-encapsulated mutant (S9Δ*cpsG*)</u>**				
	**# of mice remaining**	**Clinical score**				**# of mice remaining**	**Clinical score**			
		**0**	**> 3**	**4-6**	**< 7**		**0**	**> 3**	**4-6**	**< 7**
1	15	0	0	4	11	15	10	5	0	0
2	4	0	0	0	4	15	15	0	0	0
3	0	-	-	-	-	15	15	0	0	0
4	0	-	-	-	-	15	15	0	0	0
5	0	-	-	-	-	15	15	0	0	0
6	0	-	-	-	-	15	15	0	0	0
7	0	-	-	-	-	15	15	0	0	0
8	0	-	-	-	-	15	15	0	0	0
9	0	-	-	-	-	15	15	0	0	0
10	0	-	-	-	-	15	15	0	0	0
